# Circulating Exosomal miR-144-3p from Crohn's Disease Patients Inhibits Human Umbilical Vein Endothelial Cell Function by Targeting *FN1*

**DOI:** 10.1155/2022/8219557

**Published:** 2022-06-02

**Authors:** Peng Qu, Xiaoran Xie, Jingshu Chi, Xiaoming Liu, Peng Liu, Ju Luo, Huan Li, Sha Cheng, Xiujuan Xia, Xiong Chen, Canxia Xu

**Affiliations:** ^1^Department of Gastroenterology, The Third Xiangya Hospital of Central South University, 410013 Changsha, Hunan, China; ^2^Hunan Provincial Key Laboratory of Uncontrollable Inflammation and Tumour, The Third Xiangya Hospital of Central South University, 410013 Changsha, Hunan, China

## Abstract

**Background:**

Crohn's disease (CD) is a chronic nonspecific inflammatory disease with unknown pathogenesis and vascular changes associated with the progression of CD. Many studies have shown that miRNAs participate in the development of CD. However, the effect of miRNAs in circulating exosomes on vascular endothelial cells in CD has not been investigated. Our study is aimed at identifying the differential miRNAs in circulating exosomes in CD and exploring their potential roles in human umbilical vein endothelial cells (HUVECs).

**Methods:**

In our study, exosomes were extracted from circulating blood to identify differential miRNAs. After in vitro transfection of HUVECs with miR-144-3p mimics and inhibitors and the corresponding controls, cell counting kit-8, wound healing, Transwell migration, and tube formation assays were performed to study the viability, migration, and angiogenesis of HUVECs. Furthermore, bioinformatics analysis was used to predict miRNA targets. Western blotting was used to determine protein expression. In addition, exogenous supplementation with the fibronectin 1 (FN1) protein rescued the effects of miR-144-3p on changes in cell function in vitro.

**Results:**

miR-144-3p was significantly increased in circulating exosomes of patients with CD compared with those in the control group. The promotion or inhibition of miR-144-3p correspondingly abolished or accelerated cell viability, migration, and angiogenesis. *FN1* is a significant target of miR-144-3p, and exogenous FN1 administration improved the function of HUVECs in vitro.

**Conclusions:**

Circulating exosomal miR-144-3p from patients with active CD contributes to vascular endothelial dysfunction by affecting the gene expression of *FN1*. These findings suggested that circulating exosomal miR-144-3p could be a potential biological marker for CD.

## 1. Introduction

Crohn's disease (CD) is an immune-mediated, chronic, nonspecific disease that has become a global concern in the 21st century, with a progressive increase in prevalence in the worldwide population [1]. CD pathogenesis is still unclear, and some studies have concluded that it is caused by abnormal mucosal immunity and intestinal epithelial dysfunction [2]. However, these conclusions do not adequately explain the complex clinical presentation and outcomes of CD.

There is growing evidence that the development of CD is closely related to altered vascular function [3]. Some vascular changes, including vasodilatory dysfunction [4, 5], thrombosis [6], and atherosclerosis [7] can be found in CD. Circulating pathogenic factors may be an important part of the vascular pathology of CD. Exosomes are important carriers of circulating mediators and have become known as extracellular vesicles with diameters of 30 nm-150 nm that exist in various body fluids, including blood, saliva, urine, and milk [8]. Exosomes mainly carry signaling molecules to other cells, thereby influencing the function of other cells and mediating intercellular communication [9]. MicroRNAs (miRNAs), which are important components of exosomes, are small noncoding RNAs of 18-25 nucleotides in size [10]. miRNAs regulate posttranscriptional expression by binding to the 3′ untranslated region (UTR) of mRNAs [11, 12]. Recently, several studies have shown that exosomes or miRNAs are involved in the pathogenesis and disease progression of CD [13]. For example, plasma miRNAs is important in predicting postoperative recurrence in patients with CD [14]. miR-23a, which is highly expressed in CD intestinal tissue, increases intestinal permeability and cytokine release by targeting tumor necrosis factor-alpha inhibitor protein [15]. Moreover, exosomes can package dsDNA to promote intestinal inflammation to worsen CD [16] . However, the role of circulating exosomal miRNAs in the pathogenesis of CD is unclear.

In the present study, miR-144-3p expression was abnormally increased in the circulating exosomes of CD patients compared with healthy controls. We hypothesize that *FN1* is a target of miR-144-3p in vascular endothelial cells. Fibronectin 1(FN1) is a macromolecular glycoprotein and an important component of the extracellular matrix [17]. FN1 is associated with various diseases, such as atherosclerosis and arthritis [18, 19], and is involved in cell adhesion, cell migration, wound healing, and maintaining cell shape [20]. In addition, FN1 also plays an important role in vascular development and ECM remodeling [21]. Notably, vascular dysfunction is evidence of vascular complications in CD, and therefore, FN1 may become an important molecule in CD.

**Figure 1:**
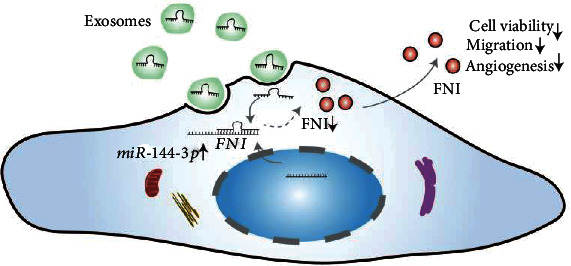
The schematic illustration of the mechanism.

We conducted this study to test the hypothesis that miR-144-3p targets the *FN1* gene to affect the viability, migration, and angiogenesis of vascular endothelial cells, (Figure 1) which may provide new ideas for the treatment of this disease.

## 2. Materials and Methods

### 2.1. Patient and Samples

After obtaining informed consent from the patients, a total of 15 patients with active CD and 15 healthy volunteers were included in the present study. See Table S1 in the Supplementary Material for demographic of Healthy controls and CD patients. The diagnosis of CD was made according to clinical, endoscopic, radiological, and histological criteria [22]. This study was approved by The Third Xiangya Hospital of Central South University Ethics Committee.

### 2.2. Exosome Isolation and Identification

Plasma from patients was centrifuged (4°C) at increasing speeds (2000 g for 20 min, 12,000 g for 70 min). The supernatant was filtered through a 0.22 *μ*m membrane and centrifuged at 4°C and 200,000 g for 120 min (Beckman Coulter Optima L-80 XP), the supernatant was discarded, and the precipitate was retained. The precipitate was resuspended in sterile phosphate-buffered saline (PBS), filtered using a 0.22 *μ*m filter, and stored at -80°C for further analysis. Morphology, particle size, and molecular markers (calnexin, Hsp70, Tsg101) of exosome were detected by transmission electron microscopy (TEM, Hitachi), nanoparticle tracking analysis (NTA; ZetaViewPMX 110), and western blot analysis, respectively.

### 2.3. Extraction of miRNA from Exosomes and Human Umbilical Vein Endothelial Cells (HUVECs)

miRNAs in exosomes or HUVECs were extracted using a miRNeasy Mini Kit (QIAGEN) according to the manufacturer's instructions. miRNAs were solubilized with 20 *μ*L of RNase-Free Water. The concentration of miRNAs was measured by a NanoDrop2000 (Thermo Fisher Scientific).

### 2.4. GO and KEGG Analyses of Target Genes of miR-144-3p

GO is a major bioinformatics tool for annotating genes and analyzing the biological processes of these genes [23]. KEGG (http://www.genome.ad.jp/kegg/) is a set of databases that expresses protein interaction networks and chemical reactions, etc., and understand and models the functional behavior of a cell or organism. [24]. The GO annotation and KEGG analyses of genes in the R package (version 3.1.0) were used as the background to map genes to the background set, and enrichment analysis was performed by using the R package clusterProfiler (version 3.14.3) to obtain the results of gene set enrichment. A minimum gene set of 5 and a maximum gene set of 5000 was set, and a *p* value < 0.05 and FDR value < 0.25 were considered statistically significant.

### 2.5. Cell Culture

HUVECs, as a very common cell type, are now widely used in vascular research. HUVECs were purchased from the Cell Resource Center (Beijing, China). The cells were maintained in Dulbecco's modified Eagle's medium/F12 (1 : 1) (Beijing, China) supplemented with 10% fetal bovine serum (Gibco) and cultured at 37°C with 5% CO_2_. The medium was changed every other day.

### 2.6. Coculture of Exosomes with HUVECs

The concentration of exosomes was determined by the BCA assay. After 24 h starvation, HUVECs were starved for 24 h in an exosome-free serum medium (Gibco). PKH-26-labeled (100 *μ*g/mL) exosomes were added to the medium with 10% exosome-free fetal bovine serum and cocultured with DAPI and phalloidin-labeled HUVECs.

### 2.7. Transfection

HUVECs were seeded into 6-well plates at 1.5 × 10^5^ cells per well and grown to about 30% confluence before being transfected. After the cells had been attached the next day, 50 *μ*M miR-144-3p mimic and 100 *μ*M miR-144-3p inhibitor were transfected into the cells using the riboFECTtm CP Transfection Kit (RiboBio) according to the manufacturer's instructions. The cells were cultured for 48 h, and then, relevant experiments were started.

### 2.8. CCK-8 Assay

To evaluate cell viability, a CCK-8 assay was used. After transfection and culture for 48 h, 10 *μ*L of CCK-8 reagent (Biosharp) was added to 100 *μ*L of culture medium, and cell viability was analyzed by measuring the optical density for each well at a wavelength of 450 nm after 4 h.

### 2.9. Wound Healing Assay

Wound healing assays were used to evaluate cell migration. The cells were seeded in 6-well plates at 1.5 × 10^5^ cells per well. After the cells were treated with the relevant reagents and reached approximately 90% confluence, cell monolayers were scratched by the tip of a 200 *μ*L pipette, washed with PBS twice gently, and cultured in a serum-free medium. Images were captured at 0 h, 24 h, 48 h, 72 h, and 96 h with at least 3 fields per well using a white light microscope. The absolute migration area ((area of initial area − an area of final area)) was calculated using ImageJ.

### 2.10. Transwell Migration Assay

For the Transwell migration assay, 24-well Transwell chambers (Corning, 8 *μ*m) were used. Briefly, a serum-free medium was added to the lower chamber, and 1 × 10^5^ cells/well were seeded in the upper chamber and cultured with a serum-free medium at 37°C with 5% CO_2_ for 8 h. Then, adherent cells in the lower chamber were fixed with 4% paraformaldehyde (Biosharp) at 4°C overnight and stained with 1% crystal violet (G-CLONE) for 30 min. We gently wiped off the cells in the upper chamber with a cotton swab. Five randomly selected fields were counted for each filter.

### 2.11. Tube Formation Assay

100 *μ*L Matrigel basement membrane matrix (Corning, 365231) was added to each well of a 96-well plate on ice, and then, the 96-well plates were incubated at 37°C and 5% CO_2_ for 1 h to solidify the Matrigel. HUVECs were transferred to 96-well plates at a density of 3 × 10^4^ cells in the medium, and tube formation was observed after 4 h. Data analysis was performed using the macroangiogenesis analyzer plug-in for ImageJ software.

### 2.12. Quantitative RT–PCR

According to the manufacturer's instructions, total RNA was extracted from HUVECs. miRNA was extracted from previously collected exosomes or HUVECs. mRNA and miRNAs were synthesized as cDNA by using the Hiscript II 1st strand cDNA Synthesis Kit and miRNA 1st-strand cDNA Synthesis kit (by the stem-loop method) (Vazyme), respectively. PCR was performed using ChamQ SYBR qPCR Master Mix for mRNA and miRNA Universal SYBR qPCR Master Mix for miRNA (Vazyme). The cycling program was set as follows: 95°C for 5 min; 50 cycles of 95°C for 10 s, 60°C for 30 s; and 95°C for 15 s, 60°C for 1 min, 95°C for 15 s, 20°C for the 20 s, *n* = 4 for each group. Gene expression was normalized to GAPDH for mRNA and U6 for miRNA, and relative quantification was calculated with the 2^−*ΔΔ*CT^ method.

miR-144-3p RT stem-loop primer is as follows: 5′-GTCGTATCCAGTGCAGGGTCCGAGGTATTCGCACTGGATACGACAGTACA-3′.

The PCR primers were as follows: FN1: 5′-CTGCAAGAGGATGGAAGGAG-3′ (forward), 5′-GGTAAATCCGGGAGGACATT-3′ (reverse); GAPDH: 5′-GGTCACCAGGGCTGCTTTTA-3′ (forward), 5′-GGATCTCGCTCCTGGAAGATG-3′ (reverse); miR-144-3p: 5′-GCGCGCGTACAGTATAGATGA-3′ (forward), 5′-AGTGCAGGGTCCGAGGTATT-3′ (reverse); and U6: 5′-CTCGCTTCGGCAGCACA-3′ (forward), 5′-AACGCTTCACGAATTTGCGT-3′ (reverse).

### 2.13. Western Blot Analysis

Total protein was extracted from the HUVECs and quantified using a BCA protein assay (Beyotime Biotechnology). Approximately 40 g of protein was loaded and separated on a 6% SDS–PAGE gel and then transferred to polyvinylidene difluoride membranes (Millipore). The membranes were blocked with 5% nonfat milk for 1 h at room temperature and then incubated with primary antibodies at 4°C overnight. Subsequently, the membranes were washed in TBS (Servicebio) with Tween-20 (Biofroxx). Next, the membranes were incubated with secondary antibodies for 2 h at room temperature. Calnexin was used as the internal control, and the bands were visualized and quantified using ImageJ software.

The primary antibodies and secondary antibodies used were as follows: Calnexin (Cell Signalling Technology), Hsp70 (Santa Cruz), Tsg101 (Santa Cruz), FN1 (Cell Signaling Technology), and horseradish peroxidase-conjugated anti-IgG secondary antibodies (Cell Signalling Technology).

### 2.14. Statistical Analysis

The data are expressed as the mean ± SD from a representative experiment. SPSS (26.0), GraphPad Prism 8.0 software, ImageJ, and R x64 4.1.1 software were used for statistical analyses. Student's *t*-test was used for comparisons between two groups. Repeated measure analysis of variance was used for comparisons among multiple groups, and *p* < 0.05 was considered statistically significant.

## 3. Results

### 3.1. Identification of Circulating Exosomes

**Figure 2:**
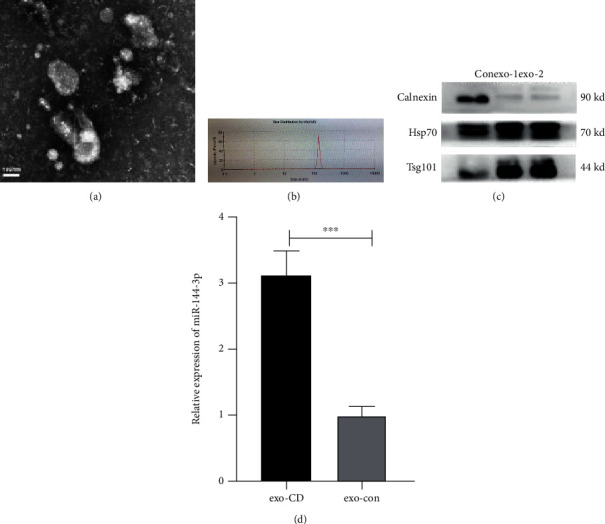
Differentially expressed miRNAs were measured in the exo-CD and exo-con groups. Morphologies of exosomes as shown by TEM (a), the size as shown by NTA (b), and specific biomarkers (HSP70 and Tsg101) as shown by western blot analysis (c). The relative expression of miR-144-3p in the exo-CD and exo-con groups, *n* = 15 for each group. ^∗∗∗^*p* < 0.001 (d).

Exosomes collected from patient plasma displayed typical exosomal features. TEM revealed a bilayer lipid membrane spheroid structure (Figure 2(a)), and the size distribution profiles ranged from approximately 100 to 150 nm (Figure 2(b)). In addition, western blot analysis revealed the expression of the exosome-specific molecular markers Hsp70 and Tsg101 but not calnexin (Figure 2(c)). These results demonstrated that exosomes were successfully extracted.

### 3.2. miR-144-3p Is Highly Increased in the Circulating Exosomes of Patients with CD

In this study, a total of thirty individuals were recruited, including fifteen CD patients and fifteen Healthy controls. After collecting and extracting their exosomes (exo-CD and exo-con), we further measured the expression of miR-144-3p. The qRT–PCR data showed that miR-144-3p expression was significantly upregulated in circulating exosomes from patients with CD (exo-CD group) (Figure 2(d)) (^∗∗∗^*p* < 0.001).

### 3.3. GO and KEGG Enrichment Analysis of Target Genes of miR-144-3p

**Figure 3:**
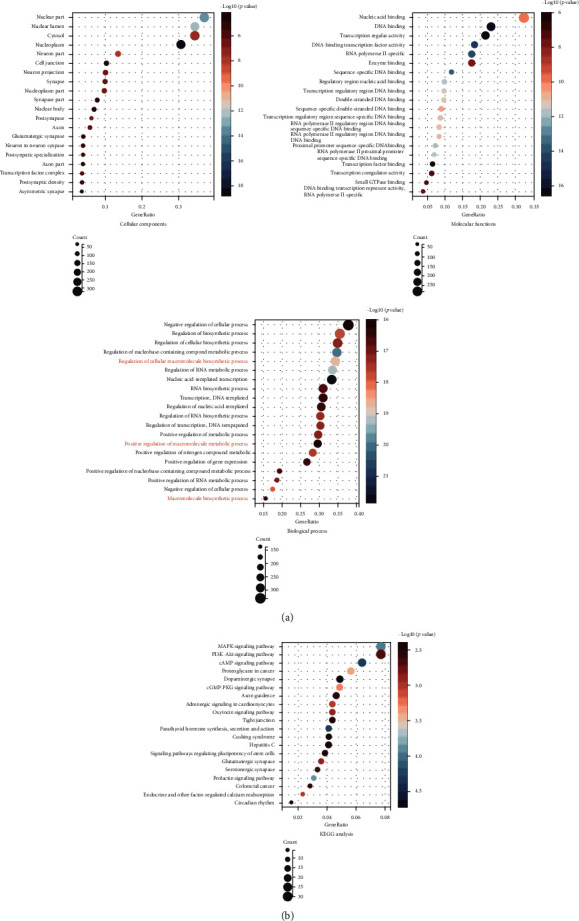
GO and KEGG analyses of the predicted target genes of miR-144-3p. The functions of these target genes are described in-depth at three levels by GO analysis including cellular components, molecular functions, and biological processes (a). KEGG pathway analysis of genes targeted to miR-144-3p (b). There was three macromolecule-related macromolecule process (red) in the biological process of GO analysis.

GO and KEGG analyses were performed to analyze the biological classification of all predicted target genes of miR-144-3p (Figures 3(a) and 3(b)). GO analysis results showed that changes in cellular components of the target genes were significantly enriched in the nuclear part, nuclear lumen, cytosol, etc. Changes in molecular function were mainly enriched in nucleic acid binding, DNA binding, transcription regulator activity, etc. Changes in the biological process were mainly enriched in negative regulation of cellular, regulation of biosynthetic and regulation of the cellular biosynthetic process, etc. These include three macromolecule-related processes. KEGG pathway analysis revealed that the target genes were mainly enriched in the MAPK signaling pathway, PI3K-Akt signaling pathway, cAMP signaling pathway, etc.

### 3.4. Uptake of Exosomes by HUVECs

**Figure 4:**
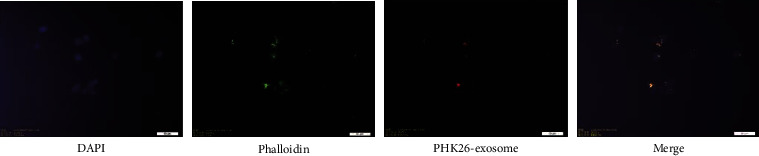
Circulating exosomes from CD patients entered into the HUVECs. Nuclei were labeled with DAPI (blue), the cytoskeleton was labeled with phalloidin (green), exosomes were labeled with PKH-26, PKH26-labeled exosomes (red) were incubated with HUVECs, and PKH26-labeled exosomes were detected inside the HUVECs as visualized using a fluorescence microscope, confirming that the exosomes entered into the cells.

After 24 hours of coculture of exosomes with HUVECs, we observed that PKH26-labeled (red) exosomes were present in HUVECs (green and blue) using fluorescence microscopy, demonstrating that circulating exosomes from CD patients can be taken up by endothelial cells (Figure 4).

### 3.5. miR-144-3p Affects the Function of Vascular Endothelial Cells In Vitro

**Figure 5:**
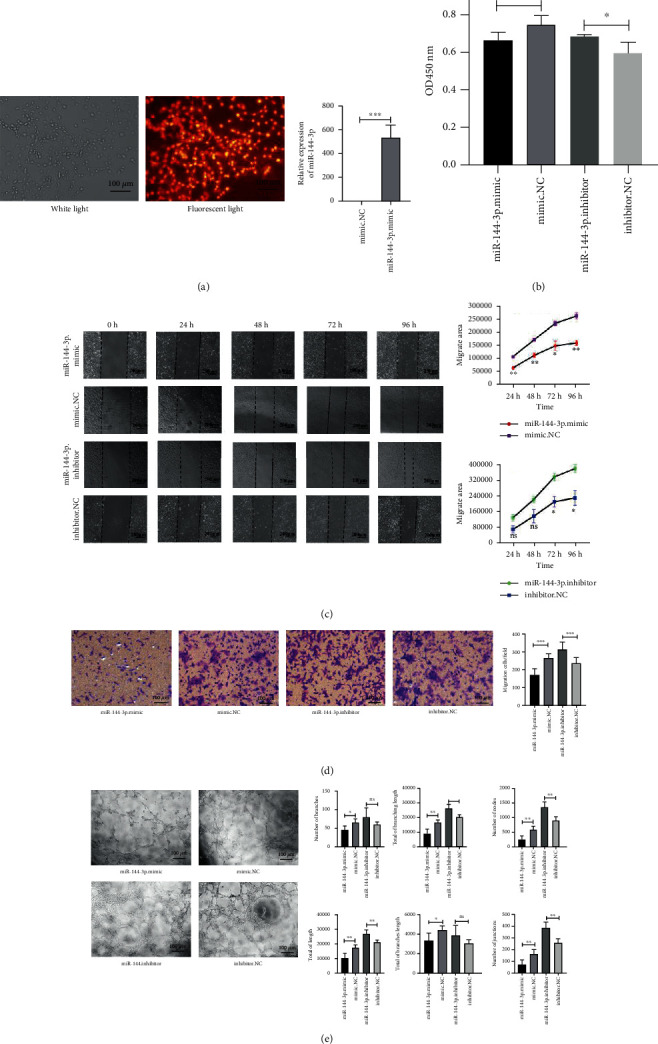
miR-144-3p affects HUVEC viability, migration, and angiogenesis. CY3 fluorescently labeled miR-144-3p-transfected HUVECs and the relative expression of miR-144-3p in miR-144-3p mimic-transfected cells and mimic NC-transfected cells (a). The viability of different transfected cells was measured by CCK-8 assays (b). Wound healing analyses of HUVECs after different transfections and representative images of the extent of cell migration into the wounded area are shown (c). The indicated cells travelled through the membrane during the Transwell invasion assay. Representative images of the Transwell assay results (d). The angiogenesis of the treated cells was evaluated by the tube formation assay (e); ^∗^*p* < 0.05, ^∗∗^*p* < 0.01, and ^∗∗∗^*p* < 0.001.

We explored the effect of miR-144-3p on HUVECs in vitro. CY3 fluorescently labeled miR-144-3p entered HUVECs successfully and miR-144-3p mimic-transfected cells showed higher expression levels of miR-144-3p than mimic. NC-transfected cells indicate that the cell models were successfully constructed. (Figure 5(a)) (*p* < 0.001). The CCK-8 assay results demonstrated that the cell viability of transfected miR-144-3p mimic cells was reduced, and the OD values showed a significant difference (*p* < 0.05) (Figure 5(b)). Wound closure was slowed in miR-144-3p mimic-transfected cells and was enhanced in miR-144-3p inhibitor-transfected cells, and the effect was time-dependent (all *p* < 0.05) (Figure 5(c)). Furthermore, we assessed the migratory capacity of HUVECs by Transwell migration assays (all *p* < 0.001) (Figure 5(d)). Finally, we also observed tube formation by HUVECs. Compared with that of the mimic NC-transfected group, the endothelial branching ability of HUVECs transfected with miR-144-3p mimic was relatively diminished (Figure 5(e)). These data indicate that miR-144-3p can regulate the invasion, migration, and angiogenesis of HUVECs.

### 3.6. Exosomal miR-144-3p Mediates Vascular Endothelial Dysfunction by Targeting the *FN1* Gene

**Figure 6:**
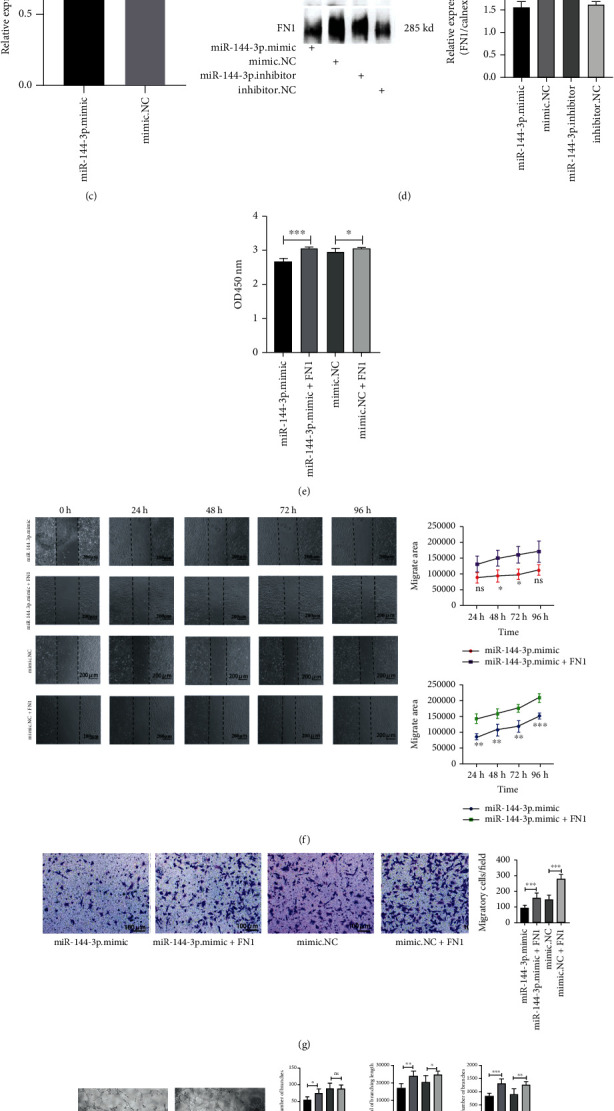
miR-144-3p inhibits HUVEC function by targeting FN1 in vitro. A Venn diagram showing the targets predicted by the four bioinformatics software programs (TargetScan 7.2, miRDB, miRmap, and miRwalk) (a). One binding site for miR-144-3p to FN1 was identified by TargetScan 7.2 website prediction (b). The mRNA expression of FN1 was unchanged (c), but FN1 protein expression was lower in miR-144-3p mimic-transfected cells (d). After exogenous addition of the FN1 protein (0.1 *μ*g/*μ*L), the viability of cells was measured by CCK-8 assays (e), and the representative images of wound healing analyses of HUVECs are shown (f). The representative images of the Transwell assay results (g), and, the images of angiogenesis in the indicated cells (h); ^∗^*p* < 0.05, ^∗∗^*p* < 0.01, and ^∗∗∗^*p* < 0.001.

According to TargetScan7.2(http://www.targetscan.org/), miRDB (http://mirdb.org/), mipmap (https://mirmap.ezlab.org/), and miRwalk (Home-miRWalk (uniheidelberg.de)) predictions, R x64 4.1.1 software was used to make a Venn diagram (Figure 6(a)), and the *FN1* gene was a target of miR-144-3p among them. TargetScan7.2 website predicted one binding site of *FN1* and miR-144-3p (Figure 6(b)). We then used qRT-PCR and western blot analysis to determine whether miR-144-3p targeted the *FN1* gene (Figures 6(c) and 6(d)); indeed, miR-144-3p affected *FN1* gene expression at the posttranscriptional level. According to the CCK-8 assay results, exogenous FN1 protein rescued the viability after transfection with miR-144-3p (^∗^*p* < 0.05) (Figure 6(e)). Wound healing assays revealed that exogenous FN1 enhanced gap closure (∗∗*p* <0.01) (Figure 6(f)). In addition, Transwell migration assays also showed that FN1 facilitated the passage of cells through small chambers (∗∗∗*p* <0.001) (Figure 6(g)). Moreover, purified FN1 protein further enhanced the diminished angiogenic capacity caused by the transfection of miR-144-3p (Figure 6(h)). Taken together, these results suggest that miR-144-3p causes vascular endothelial cell dysfunction by affecting *FN1* gene expression.

## 4. Discussion

Differential miRNAs have been reported in intestinal mucosal tissue or the serum of CD patients [25, 26]. However, the miRNAs expression profiles of circulating exosomes remain poorly characterized. A study showed that miR-144 is significantly upregulated in the intestinal mucosa in active CD patients compared with healthy controls [27]. Another study also showed that miR-144 is increased in Crohn's lesion tissue compared to healthy intestinal mucosa tissue [28]. Fortunately, we also found that miR-144-3p is similarly different in circulating exosomes in CD patients relative to healthy controls. After several experiments, we also detect some other different miRNAs in patient and healthy human exosomes. miR-144-3p have relatively large differences among these miRNAs, and we are interested in miR-144-3p. To the best of our knowledge, we are the first to find a significant increase in circulating exosomal miR-144-3p in patients with CD, suggesting that miR-144-3p in circulating exosomes may be a new molecular marker for the diagnosis of CD. Vascular endothelial cells are the first to come into contact with exosomes in blood, so we investigated whether circulating exosomal miR-144-3p affected vascular endothelial cells, which may are involved in the pathogenesis of CD.

In the present study, we performed GO and KEGG enrichment analyses to explore the biological functions and signaling pathways in which all predicted target genes were enriched. GO enrichment analysis revealed that the most significant modules were mainly enriched in macromolecule-related processes, FN1, a macromolecule, is one of the targets of miR-144-3p, and this theory is consistent with our results.

To further elucidate the mechanisms of miR-144-3p in vascular endothelial cells, HUVECs were transfected with miR-144-3p mimics, mimic NC, miR-144-3p inhibitor, and inhibitor NC. We found that miR-144-3p could inhibit the viability, migration, and tube formation of HUVECs. Similar to the role of miR-144-3p in colorectal cancer cells, Zhou et al. also reported that the downregulation of miR-144-3p promoted the proliferation and migration of colorectal cancer cells [29]. In addition, Li et al. also found that upregulating miR-144-3p inhibited the proliferation, migration, and invasion abilities of hepatocellular carcinoma cells [30]. Moreover, one study revealed that circulating exosomal miR-144-3p indirectly impaired the mobilization of endothelial progenitor cells and thus inhibited angiogenesis [31]. The conclusions drawn in our study could explain vascular endothelial dysfunction in CD, such as atherosclerosis [7]. In addition, these results also provide a basis for the impaired intestinal barrier in CD [32].

Some studies have suggested that miR-144-3p affects papillary thyroid cancer cell migration and invasion by targeting the *FN1* gene, and its relevance was demonstrated by using a double luciferase reporter [33, 34]. However, it is not clear whether miR-144-3p can regulate the function of HUVECs or the role of the FN1 protein. Moreover, four bioinformatics software programs (TargetScan 7.2, miRDB, miRmap, and miRwalk) predicted that the target gene of miR-144-3p. Among the 108 predicted target genes, the FN1 protein was highly expressed in HUVECs and is associated with the function of HUVECs. We next examined the correlation of *FN1* and miR-144-3p expression in cell models.

FN1 is a large protein with a relatively large transcript, and it was difficult for us to construct overexpression cell lines by plasmid or viral transfection *FN1*, which is synthesized mainly in hepatocytes [35], is secreted from the cell, and is widely found in the extracellular matrix and plasma. As shown in previous studies, Shan et al. reported that miR-17 inhibited cell adhesion and proliferation by repressing *FN1* expression, and purified FN1 protein was used to confirm the effect on *FN1* expression in rescue experiments [36]. Indeed, our studies showed that exogenous FN1 significantly abrogated the effects of miR-144-3p. Although we provide evidence to support a regulatory role of miR-144-3p on *FN1* gene expression, the exosomes are enriched with other differential proteins, lipids, and nucleic acid molecules [37]; we also acknowledge that other substances in exosomes may affect vascular endothelial alterations.

Current treatments for CD include immunosuppressants, biological therapies, and nutritional therapy [38], which are only able to control the disease, relieve patient symptoms, and improve patient quality of life but hardly achieve a cure. miR-144-3p has antivascular effects and could be used as a potential therapeutic agent. With advances in exosome research over the years, exosomes have been shown to act as biological vesicles capable of delivering relevant therapeutic agents, such as antiangiogenic agents, for the treatment of CD [39].

However, this research still has some limitations. First, we did not use animal models for our experiments, which was a drawback due to the difficulty of making models and the variability of the species. Second, only 30 plasma samples were collected for this study; therefore, the small sample size and other differences between patients may influence the results.

## 5. Conclusions

These findings will help us to determine whether miR-144-3p in circulating exosomes can serve as a new biological marker for CD and may provide a novel mechanism for the interpretation of vascular dysfunction in active CD. Further research is required on the use of exosome-delivered miRNAs for the targeted treatment of CD.

## Data Availability

The (data type) data used to support the findings of this study are available from the corresponding author upon request.

## Abbreviations

CD: Crohn's disease
HUVECs: Human umbilical vein endothelial cells
FN1: Fibronectin 1
miRNAs: MicroRNAs
UTR: Untranslated region
PBS: Phosphate-buffered saline
TEM: Transmission electron microscopy
NTA: Nanoparticle tracking analysis
exo-CD: Exosome of CD
exo-con: Exosome of control
exo1: Exosome 1
exo2: Exosome 2.

## Supplementary Materials

Supplementary Materials Table S1: Demographic of healthy controls and CD patients.
