# Supplementary material for: Circulating Exosomal miR-144-3p from Crohn's Disease Patients Inhibits Human Umbilical Vein Endothelial Cell Function by Targeting FN1
Source: Dis Markers. 2022 Jun 2;2022:8219557. doi: 10.1155/2022/8219557 (PMC9184168; doi:10.1155/2022/8219557)
Supplement: Supplementary Materials — Table S1: Demographic of healthy controls and CD patients. [file 8219557.f1.pdf]

Table S1 Demographic of Healthy controls and CD patients.

	Healthy control	CD	*p* value
Number	15	15	
Male/Female	12/3	13/2	1.0
Age(years,mean±SD)	33.5±11.1	31.7±10.1	0.73
Marriage, n(%)	11(73.3%)	10(66.7%)	1.0
Height(m, mean±SD)	1.68±0.07	1.68±0.43	0.99
Weight(kg, mean±SD)	56.2±11.0	54.6±9.1	0.67
BMI(kg/m^2)	20.6±4.2	18.9±2.3	0.18
Smoking, n(%)	6(40%)	6(40%)	1.0
Alcohol consumption,n(%)	2(13.3%)	3(20%)	1.0
